# Horizontal Gene Transfer of a ColV Plasmid Has Resulted in a Dominant Avian Clonal Type of *Salmonella enterica* Serovar Kentucky

**DOI:** 10.1371/journal.pone.0015524

**Published:** 2010-12-22

**Authors:** Timothy J. Johnson, Jessica L. Thorsness, Cole P. Anderson, Aaron M. Lynne, Steven L. Foley, Jing Han, W. Florian Fricke, Patrick F. McDermott, David G. White, Mahesh Khatri, Adam L. Stell, Cristian Flores, Randall S. Singer

**Affiliations:** 1 Department of Veterinary and Biomedical Sciences, University of Minnesota, St. Paul, Minnesota, United States of America; 2 Department of Biological Sciences, Sam Houston State University, Huntsville, Texas, United States of America; 3 Division of Microbiology, National Center for Toxicological Research, U.S. Food and Drug Administration, Jefferson, Arkansas, United States of America; 4 Institute for Genome Sciences, University of Maryland School of Medicine, Baltimore, Maryland, United States of America; 5 Division of Animal and Food Microbiology, Office of Research, Center for Veterinary Medicine, U.S. Food and Drug Administration, Laurel, Maryland, United States of America; University of Hyderabad, India

## Abstract

*Salmonella enterica* continues to be a significant cause of foodborne gastrointestinal illness in humans. A wide variety of *Salmonella* serovars have been isolated from production birds and from retail poultry meat. Recently, though, *S. enterica* subsp. *enterica* serovar Kentucky has emerged as one of the prominent *Salmonella* serovars isolated from broiler chickens. Recent work suggests that its emergence apparently coincides with its acquisition of a ColV virulence plasmid. In the present study, we examined 902 *Salmonella* isolates belonging to 59 different serovars for the presence of this plasmid. Of the serovars examined, the ColV plasmid was found only among isolates belonging to the serovars Kentucky (72.9%), Typhimurium (15.0%) and Heidelberg (1.7%). We demonstrated that a single PFGE clonal type of *S*. Kentucky harbors this plasmid, and acquisition of this plasmid by *S*. Kentucky significantly increased its ability to colonize the chicken cecum and cause extraintestinal disease. Comparison of the completed sequences of three ColV plasmids from *S*. Kentucky isolated from different geographical locales, timepoints and sources revealed a nearly identical genetic structure with few single nucleotide changes or insertions/deletions. Overall, it appears that the ColV plasmid was recently acquired by a single clonal type *S*. Kentucky and confers to its host enhanced colonization and fitness capabilities. Thus, the potential for horizontal gene transfer of virulence and fitness factors to *Salmonella* from other enteric bacteria exists in poultry, representing a potential human health hazard.

## Introduction

ColV plasmids are virulence plasmids that have been associated with extraintestinal pathogenic *Escherichia coli*, or ExPEC [1], [2]. This *E. coli* pathotype causes a number of extraintestinal diseases of humans and animals, and isolates belonging to this pathotype are genetically diverse [3]. ColV plasmids have been most strongly associated with avian pathogenic *E. coli* (APEC) and neonatal meningitis-associated *E. coli* (NMEC), which are subpathotypes of ExPEC causing avian colibacllosis and human neonatal meningitis, respectively [4], [5]. ColV plasmids are so named for their ability to produce multiple bacteriocins, including colicin V (ColV) [6]. They also contain a highly conserved pathogenicity-associated island (PAI) that encodes for a number of virulence factors, including multiple iron acquisition and transport mechanisms, serum survival proteins, avian-like hemolysins, outer membrane proteins, adhesins, and autotransporters [7], [8]. These plasmids have been shown to play an important role in the virulence of ExPEC [1], [9], [10], [11].

A *Salmonella* genome sequencing effort involving multidrug-resistant and drug susceptible strains from serovars of human and animal health importance was recently completed via a collaboration between U.S. FDA and the J. Craig Venter Institute. This work revealed that one of these strains, *Salmonella enterica* subsp. *enterica* serovar Kentucky strain CVM29188, contained a ColV plasmid in its genome [12]. This was unexpected because it was the first evidence of a ColV plasmid occurring among *Salmonella* isolated directly from the environment (in this case, from retail poultry meat). Fricke et al. described the plasmid complement of this strain, and the ColV plasmid possessed by strain CVM29188 was highly similar to those of APEC strains previously described [12]. The authors also screened 287 *S*. Kentucky isolates from animal, human, and retail meat sources for the prevalence of ColV plasmid-associated genes, and found that 74% of the isolates from chicken samples tested positive for these genes [12].

The prominent *Salmonella* serovars found among poultry include Kentucky, Heidelberg, and Typhimurium, of which the latter two are considered important human pathogens. Interestingly, evidence from the Center for Disease Control's Foodborne Diseases Active Surveillance Network, or FoodNet, suggests that *S*. Kentucky has only recently emerged as a dominant serovar of poultry. In fact, the fraction of *S*. Kentucky isolated from chickens has increased steadily over the past decade, ranging from 25% in 1997 to nearly 50% in 2006 [13], [14]. Other researchers have reported similar findings in prevalence studies involving chickens. The apparent emergence of ColV plasmid-containing *S*. Kentucky clones among poultry led to the hypothesis that this plasmid might play a role in *S*. Kentucky's ability to persist in the poultry farm environment and colonize the avian host. Here, we tested this hypothesis utilizing several *in vitro* and *in vivo* approaches to examine a potential role for the ColV plasmid in the colonization, fitness, and virulence capabilities of *S*. Kentucky strains in chickens. Additionally, we screened a large collection of *Salmonella* isolates from human, avian, and environmental sources to determine the extent of the dissemination of ColV plasmids among avian-source *Salmonella* strains. Furthermore, DNA sequencing and analysis was performed on two additional *S*. Kentucky ColV plasmids to determine their genetic relatedness with one another and other sequenced ColV plasmids.

## Results

### The ColV plasmid is highly prevalent among a single *S*. Kentucky pulsed-field gel electrophoresis (PFGE) clonal type isolated from chickens and the poultry farm environment

Nine hundred and two *Salmonella* isolates representing 59 different serovars were examined for the presence of ColV plasmid-associated genes via multiplex PCR (Table 1). Of these, ColV plasmid-associated genes were detected among the Kentucky, Typhimurium and Heidelberg *Salmonella* serovars (Table S1). Of the 119 *S*. Heidelberg analyzed, 2 (1.7%) contained *iutA* and *cvaC* but not *iss*. Of the 100 *S*. Typhimurium analyzed, 15 (15.0%) possessed ColV plasmid-associated genes and several gene combinations were observed, with the most prevalent profiles being *iss*-*iutA*-*eitC*-*iroN*-*cvaC* and *iss*-*iutA*-*iroN*-*cvaC*. Of the 293 *S*. Kentucky analyzed, 213 (72.7%) contained all of the expected genes sought as compared to pCVM29188_146 (*iutA*, *iss*, *iroN*, and *cvaC*). None of the *S*. Kentucky isolates examined differed in the profile of positive genes within the multiplex panel studied. The overall proportion of *S*. Kentucky isolates that possessed ColV plasmid-associated genes was higher in farm samples (85.3%) than in processing plant samples (38.0%; p<0.0001) (Table S2). PFGE analysis performed on a subset of 71 *S*. Kentucky isolates generated 14 distinct PFGE profiles based on differential banding patterns. Overall, a single PFGE profile emerged as the prominent profile among farm and processing plant isolates (subsequently referred to as the chicken PFGE profile), with 100% of the ColV^+^ isolates belonging to this profile.

**Table 1:** Multiplex PCR primers used for determining ColV plasmid prevalence.

Gene	Primer	Description	Product (bp)	Sequence (5′ to 3′)
*invA*	F	*Salmonella* internal control	244	ACAGTGCTCGTTTACGACCTGAAT
	R			AGACGACTGGTACTGATCGATAAT
*iutA*	F	Aerobactin receptor gene	302	GGCTGGACATCATGGGAACTGG
	R			CGTCGGGAACGGGTAGAATCG
*iss*	F	Serum survival gene	325	CAGCAACCCGAACCACTTGATG
	R			AGCATTGCCAGAGCGGCAGAA
*eitC*	F	Putative iron transport system gene	380	CAGCAGCGCTTCGGACAAAATCTCC
	R			TTCCCCACCACTCTCCGTTCTCAAAC
*iroN*	F	Salmochelin siderophore system gene	553	AATCCGGCAAAGAGACGAACCGCCT
	R			GTTCGGGCAACCCCTGCTTTGACTTT
*cvaC*	F	Colicin V synthesis gene	678	CACACACAAACGGGAGCTGTT
	R			CTTCCCGCAGCATAGTTCCAT
*csgA*	F	*E. coli* internal control	200	ACTCTGACTTGACTATTACC
	R			AGATGCAGTCTGGTCAAC

**Figure 1:**
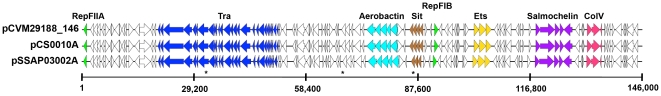
Linear genetic maps of three sequenced ColV plasmids from *S*. Kentucky from different geographical sources. Arrows indicate predicted coding regions. Colored arrows depict replication genes (green), F transfer region (dark blue), aerobactin siderophore system (light blue), Sit and Ets iron transport systems (brown and yellow), salmochelin siderophore system (purple) and ColV operon (pink). SNPs or deleted regions are indicated with a “*” below the map. Scale is given in base pairs.

DNA sequencing was performed on ColV plasmids from two *S*. Kentucky strains isolated from cloacal swabs in different U.S. states and at different timepoints, and these sequences were compared to one another and to the completed sequence of pCVM29188_146 from an *S*. Kentucky isolate of retail poultry [12]. These three plasmids shared remarkable nucleotide similarity, with a 804-bp deletion of the *sitA* gene in pSSAP03002A_146 and three non-IS-associated single nucleotide polymorphisms being the only key differences between the plasmids (Table 2 and Fig. 1).

**Table 2:** Comparison of single nucleotide polymorphisms or deleted regions within the three sequenced *S*. Kentucky ColV plasmids.

Coordinates:	pCVM29188_146146,811 bp	pCS0010A146,811 bp	pSSAP03002A146,002 bp	Gene
88837-89641	present	present	deleted	*sitA*
32087	T	C	T	*traG*
67699	C	T	C	NC^A^
90000	T	T	C	IS1
90003	G	G	C	IS1
90006	T	T	C	IS1
90015	T	T	G	IS1
101030	C	T	C	IS1203
113681	C	C	T	IS2

A NC  =  non-coding region.

### Strain- and serovar-specific differences exist in *Salmonella* growth capabilities in serum and low iron media

**Figure 2:**
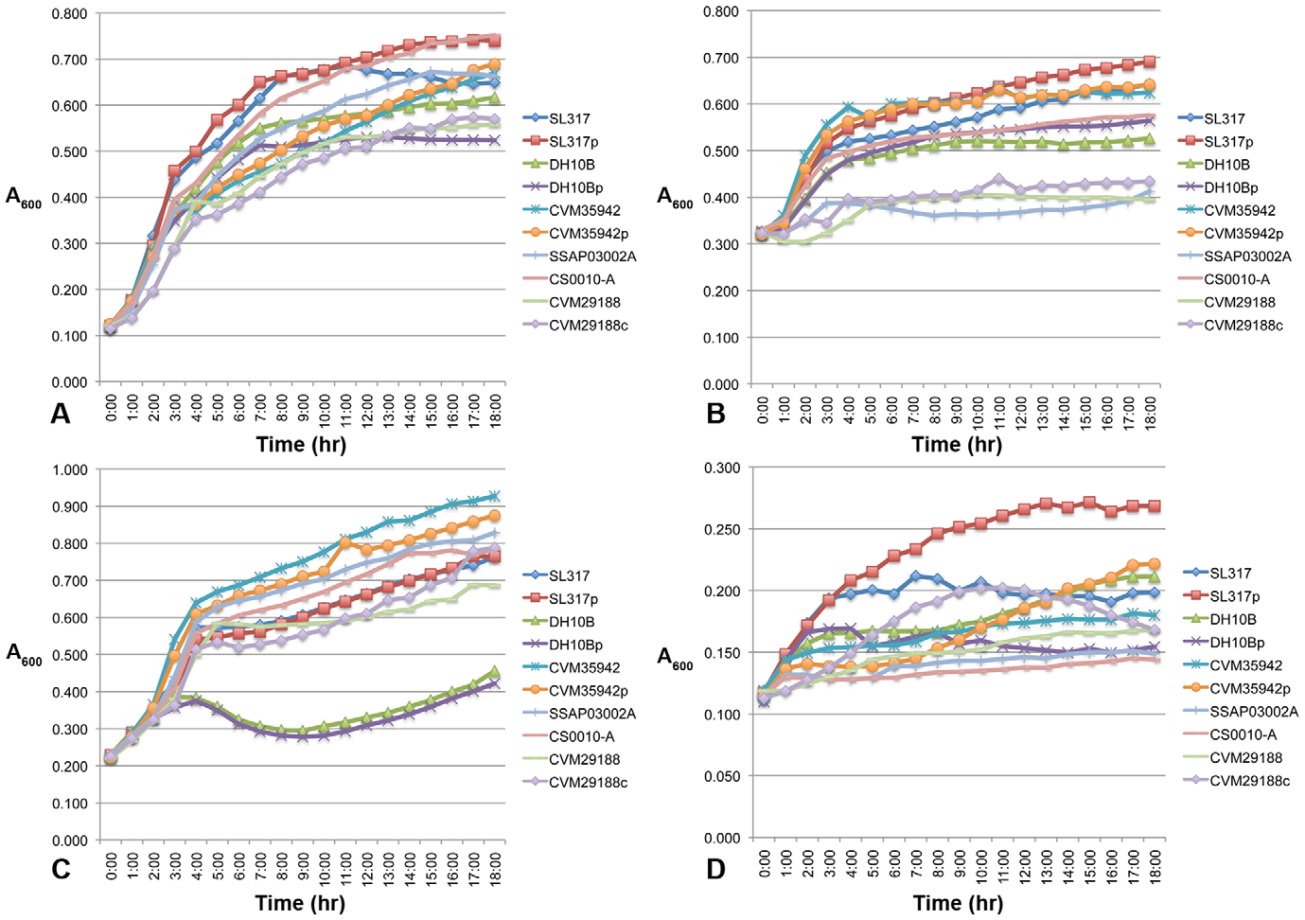
Growth curves of selected isolates in (A) LB broth, (B) human serum, (C) chicken serum, and (D) LB broth +200 uM 2,2′-dipyridyl. Strains with “p” refer to the transconjugant of a strain containing pCVM29188_146, while strains with “c” refer to a wild type strain cured of its ColV plasmid.

A collection of ColV plasmid-containing and plasmid-lacking *Salmonella* isolates (Table 3) was used to test their abilities to grow in LB broth, LB broth supplemented with 200 uM 2,2′-dipyridyl (low iron media), 50% human serum in LB broth, and 100% chicken serum over an 18-hour period (Fig. 2). In LB broth, no differences were observed between plasmid-containing and plasmid-lacking strains, but *S*. Newport strain SL317 grew better than other strains over the 18-hr time period. In 50% human serum, *S*. Newport SL317p fared slightly better than *S*. Newport SL317 while no other differences were observed between plasmid-containing and plasmid-lacking strains. *S*. Newport SL317 and *S*. Kentucky CVM35942 grew the best in human serum, while *S*. Kentucky strains CVM29188 and SSAP03002A performed poorly. In 100% chicken serum, no differences were observed between plasmid-containing and plasmid-lacking strains. However, *S*. Kentucky strains CVM35942, CS0010-A, and SSAP03002A performed better than other strains in this medium. In low iron media, the isolates generally grew poorly, except that *S*. Newport SL317p grew better than its plasmid-lacking counterpart SL317.

**Table 3:** Bacterial strains used in this study.

Strain name	Serovar/serogroup	Source	Traits	Use in this study
CVM29188	Kentucky	Chicken breast	ColV+	FPM^A^
CVM29188c^B^	Kentucky	Plasmid-cured derivative of CVM29188	ColV−	F
CVM35942	Kentucky	Human	ColV−	CF
CVM35942p^C^	Kentucky	Transconjugant of CVM35942	ColV+	CF
SL317	Newport	Human	ColV−	CF
SL317p	Newport	Transconjugant of SL317	ColV+	CF
CS0010-A	Kentucky	Chicken cloaca	ColV+	CP
SSAP03002A	Kentucky	Chicken cloaca	ColV+	CP
APEC O1	O1	Avian colibacillosis	ColV+	F
APEC O2	O2	Avian colibacillosis	ColV+	F
APEC O2c	O2	Plasmid-cured derivative of APEC O2	ColV−	F
DH10B	NA	Laboratory strain	ColV−	F
DH10B + pCVM29188_146	NA	Transconjugant containing ColV plasmid from CVM29188	ColV+	F
DH10B + pAPEC-O2-ColV	NA	Transconjugant containing plasmid from APEC O2	ColV+	F

A C  =  colonization studies; F  =  extraintestinal fitness studies; P  =  comparative plasmid sequencing; M  =  PCR prevalence studies.

B c  =  refers to wild type strain cured of its ColV plasmid.

C p  =  refers to transconjugant of strain containing pCVM29188_146.

### Acquisition of the ColV plasmid by *S*. Kentucky increases its colonization ability in chickens

**Figure 3:**
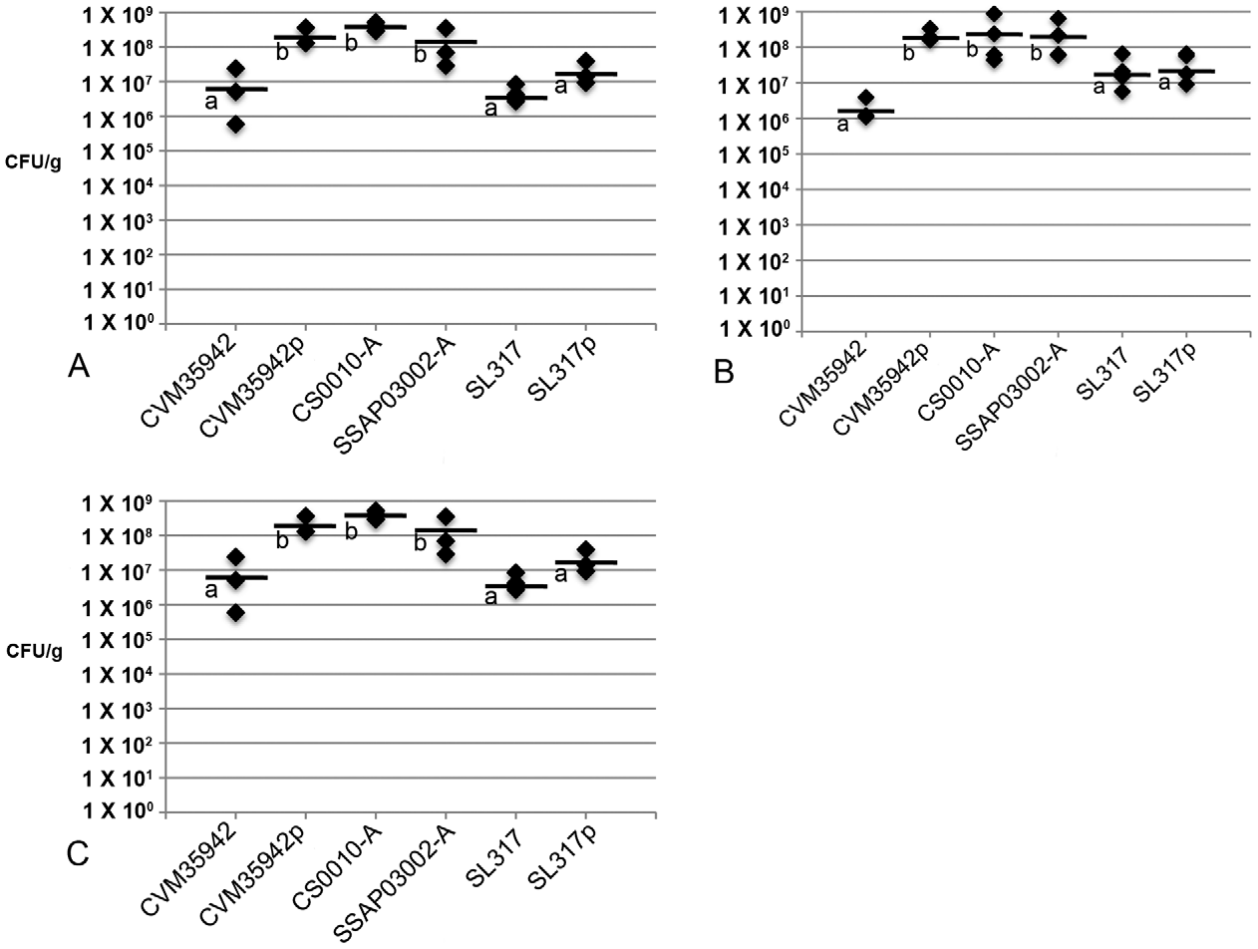
Relative colonization abilities of *S*. Kentucky strain CVM35942 and its plasmid-containing transconjugant, *S*. Kentucky strains CS0010-A and SSAP03002-A, and *S*. Newport strain SL317 and its plasmid-containing transconjugant. Four samples each were collected on days 3 (A), 7 (B), and 14 (C) post-inoculation. Bacterial counts are depicted in CFU/g tissue. Letters alongside each mean depicts significantly different groups (p<0.05) calculated using ANOVA in conjunction with Tukey's range test. Strains with “p” refer to the transconjugant of a strain containing pCVM29188_146.

**Figure 4:**
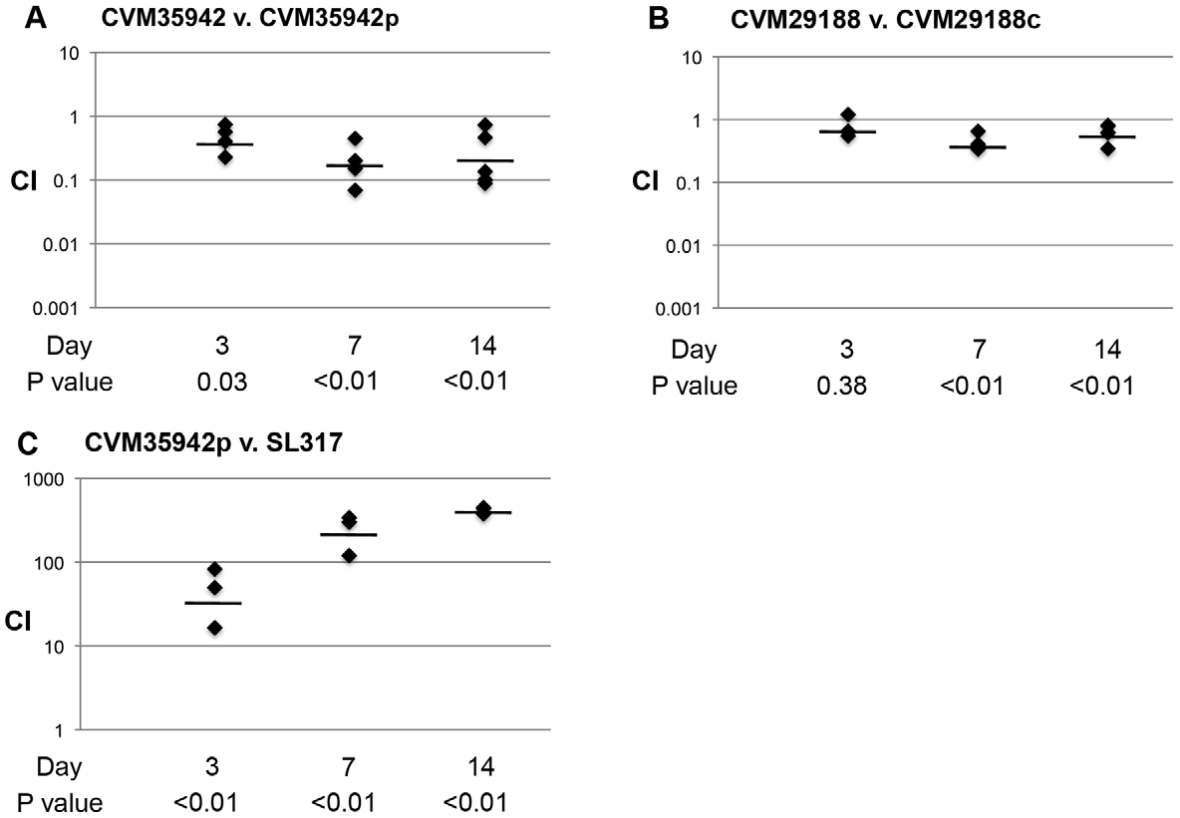
Chicken competition colonization model using plasmid-containing and plasmid-lacking strains. (A) competes plasmid-containing and plasmid lacking *S*. Kentucky CVM35942; (B) competes plasmid-containing and plasmid lacking *S*. Kentucky CVM29188; (C) competes *S*. Kentucky CVM35942p with *S*. Newport SL317. Competition Index (CI) values are presented so that a CI value >1 means that strain 1 outcompeted strain 2, and a CI value <1 means that strain 2 outcompeted strain 1. P values shown below Day were calculated relative to 1 using a Student's *t-*test. Strains with “p” refer to the transconjugant of a strain containing pCVM29188_146, while strains with “c” refer to a wild type strain cured of its ColV plasmid.

A collection of ColV plasmid-containing and plasmid-lacking *Salmonella* isolates (Table 3) was used to test their abilities to colonize specific-pathogen-free (SPF) chickens. The isolates were compared in a relative colonization model (Fig. 3) and a competition model (Fig. 4). In the relative colonization model, plasmidless *S*. Kentucky strain 35942 belonging to the chicken PFGE profile was compared to its ColV plasmid-containing transconjugant. Similarly, *S*. Newport strain SL317, a human isolate, was compared to its ColV plasmid-containing transconjugant. For comparison purposes, two additional ColV^+^ wild type *S*. Kentucky strains from broiler chickens (CS0010-A and SSAP03302-A) were also compared, both belonging to the chicken PFGE profile. On all days sampled over a two-week period post-inoculation, plasmid-containing strain CVM35942p colonized significantly better (p<0.05) than its plasmid-lacking counterpart. CVM35942p also colonized at similar levels to the other wild type ColV^+^ *S*. Kentucky strains belonging to the same chicken PFGE profile. These plasmid-containing *S*. Kentucky strains colonized significantly better (p<0.05) than either of the *S*. Newport strains tested. The *S*. Newport strain SL317p containing the ColV plasmid colonized better than plasmid-lacking SL317 at all timepoints, but these differences were not statistically significant. Spleen tissues were also cultured from all birds tested, but no bacteria were recovered in any of the samples (data not shown).

In the competition model, three comparisons were made over two weeks post-inoculation (Fig. 4). Strain CVM35942p significantly outcompeted its plasmid-lacking counterpart at all timepoints tested (2- to 5-fold). In contrast, *S*. Kentucky strain CVM29188 containing the ColV plasmid was outcompeted by its plasmid-cured counterpart at two of three timepoints (approximately 1.5- to 2-fold). When *S*. Kentucky strain CVM35942p was competed against *S*. Newport strain SL317, it greatly outcompeted SL317 at all timepoints tested (50- to 421-fold).

### Acquisition of the ColV plasmid by *S*. Kentucky enables its extraintestinal virulence in chickens

**Figure 5:**
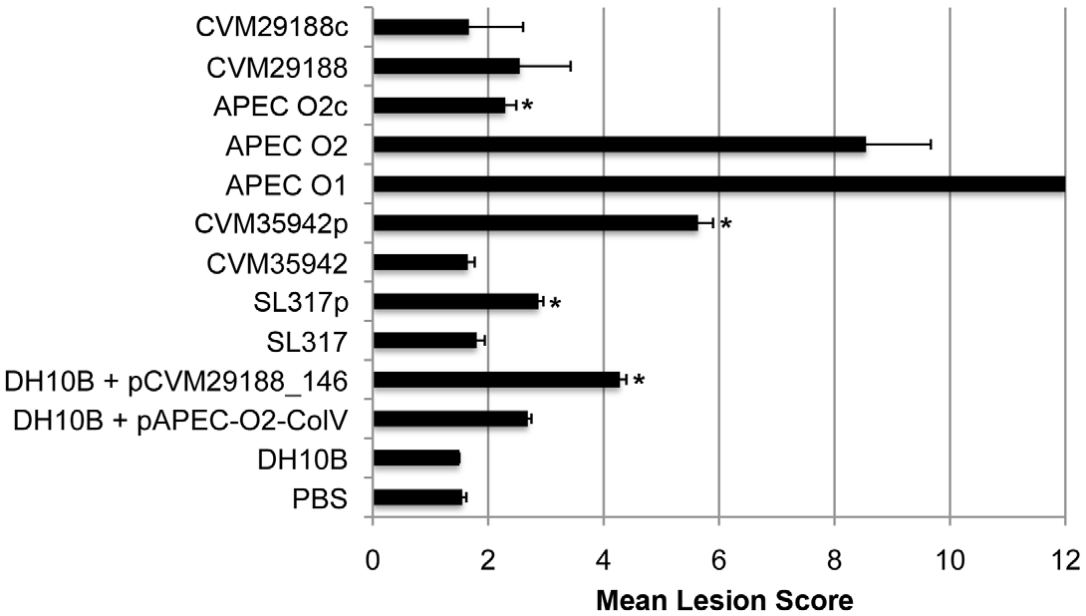
Subcutaneous model of extraintestinal disease in broiler chicks using selected plasmid-containing and plasmid-lacking strains. An asterisk next to the mean lesion score indicates a significant change (p<0.05) as compared to a strain's plasmid-containing or plasmid-lacking counterpart. Results shown are the combination of two independent trials. Strains with “p” refer to the transconjugant of a strain containing pCVM29188_146, while strains with “c” refer to a wild type strain cured of its ColV plasmid.

To study the contribution of the *S*. Kentucky ColV plasmid to extraintestinal fitness/virulence, two strains belonging to differing PFGE profiles of *S*. Kentucky were used (CVM29188 and CVM35942), along with *E. coli* strains APEC O1 [15], APEC O2 [16], [17], and DH10B; *S*. Newport strain SL317; and transconjugants or plasmid-cured derivatives thereof (Table 3 and Fig. 5). In all cases, acquisition of the ColV plasmid resulted in an increased mean lesion score, whereas plasmid curing resulted in a decreased lesion score. These changes were statistically significant (p<0.05 using the Wilcoxon signed-rank test) for APEC O2 v. APEC O2c, CVM35942 v. CVM35942p, SL317 v. SL317p, and DH10B v. DH10B + pCVM29188_146.

## Discussion

*S*. Kentucky have been increasingly isolated from hatchery chicks [18], [19], commercial broiler and layer flocks [18], [20], [21], [22], commercial broiler environments [23], broiler processing plants [24], [25], [26], and retail poultry products [27], [28]. In addition to the association of *S*. Kentucky with poultry, it has also been isolated from other environments and reservoirs. For example, *S*. Kentucky has recently been reported as a predominant serovar in pet dogs [29], and there have been recent reports of *S*. Kentucky's implication with human disease [30], [31], [32] and an increase in the reported cases of *S*. Kentucky-associated human disease by the CDC [33], [34]. Certainly, the increased association of *S*. Kentucky with human disease, the already-established association of *S*. Heidelberg and *S*. Typhimurium with human disease, and their prominence in poultry warrants further efforts aimed at understanding the basis for their apparent persistence in the poultry farm environment.

**Figure 6:**
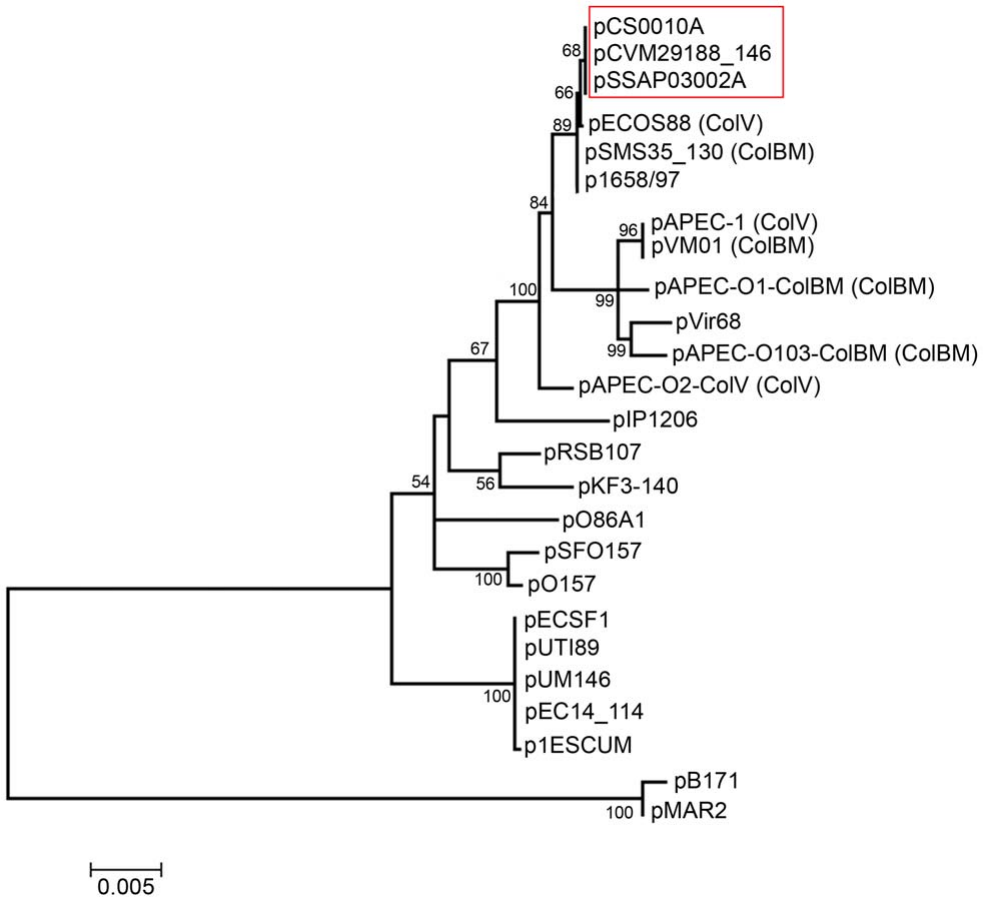
Evolutionary relationships of 25 sequenced RepFIB/FIIA plasmids conducted in MEGA4 [48]. Evolutionary history was inferred using the *repA* and *repA1* concatenated gene sequences from each plasmid totaling 1,832 bp. Bootstrap consensus tree was constructed from 1,000 replicates with percentages greater than 50% shown at branches. The tree is drawn to scale to represent inferred evolutionary distances computed with the Maximum Composite Likelihood method depicted as base substitutions per site. *S*. Kentucky ColV plasmids are boxed in red.

Here, we found that nearly 73% of the *S*. Kentucky analyzed possessed a ColV plasmid. Further analysis of these plasmid-containing isolates with PFGE revealed that all of the plasmid-containing isolates examined belonged to a single PFGE *Xba*I restriction profile. Comparison of three sequenced *S*. Kentucky ColV plasmids, whose host strains differed in isolation both geographically and temporally, revealed that they were essentially identical to one another (Fig. 1). This is particularly striking, considering that the ColV plasmids have been shown to be extremely heterogeneous in their genetic structure even among closely related bacteria [7], [8], [12], [36]. The presence of nearly identical plasmids in multiple isolates belonging to a single PFGE profile suggest that the ColV plasmid has been transferred to *S*. Kentucky in a single rare event, and that clonal expansion has subsequently occurred resulting in a prominent clonal type of *S*. Kentucky harboring a ColV plasmid in poultry. An alignment of the nucleotide sequences of the replication genes from these plasmids with those of other sequenced IncFIB/FIIA plasmids confirms that they are highly similar to other sequenced ColV plasmids from *E. coli* and likely share a recent common ancestry (Fig. 6). It is speculative in nature to discuss how or when such a plasmid transfer occurred, but it is certainly plausible that this transfer could have occurred between APEC and *S*. Kentucky within the bird, or within the poultry environment. The fact that *S*. Kentucky has emerged in poultry worldwide suggests a common source for these isolates, such as from the hatchery.

We also found the presence of ColV plasmid-associated genes among the *S*. Heidelberg and *S*. Typhimurium isolates examined, albeit at a much lower prevalence. These isolates differed from the *S*. Kentucky isolates in their plasmid-associated gene profiles, suggesting that variants of the ColV plasmid have been introduced into these *Salmonella* serovars on other occasions. Further work is necessary to determine the impact of the acquisition of these plasmids on their hosts. Our experimental data suggest that the same plasmid can have a much different effect on different *Salmonella* hosts that acquire it, even among strains belonging to the same serovar. Therefore, the results presented here for *S*. Kentucky may not necessarily apply to other strains and/or serovars. It should also be acknowledged that some of the genes tested, including *iutA* and *iroN*, could also be located within the bacterial chromosome. Therefore, it is possible that not all of the isolates that were positive for these genes actually possessed a ColV plasmid. However, the presence of ColV-plasmid specific genes in these same isolates strongly suggests that they do indeed carry the plasmid.

Results of the colonization experiments suggest that the *S*. Kentucky ColV plasmid enhances the ability of its host strain to colonize the chicken and outcompete other bacteria within the chicken cecum. Enhancement of colonization conferred by the ColV plasmid is a phenomenon previously observed for *E. coli* in both the human and avian gastrointestinal tracts [1], [35]. This property is likely attributed to the presence of afimbrial adhesins and colicins on this plasmid [36], allowing for increased adhesion and the ability to outcompete other bacteria in the gut, respectively. Despite an apparent role for the ColV plasmid in *S*. Kentucky's enhanced colonization capabilities, it is also evident that chromosomal traits must contribute to *S*. Kentucky's success in poultry. Therefore, more work is needed to dissect the colonization mechanisms of this emergent *S*. Kentucky clone.

In addition to enhanced colonization, acquisition of the ColV plasmid by *S*. Kentucky also increased its virulence in an extraintestinal (subcutaneous) model of infection. We chose this model because most *Salmonella* are typically associated with gastroenteritis and are not expected to cause disease as extraintestinal pathogens; therefore, an increase in lesion score would indicate an increase in overall extraintestinal fitness of the strain examined. Acquisition of the ColV plasmid by *S*. Kentucky 35942 and *S*. Newport SL317 significantly increased their mean lesion scores, and did so strikingly in the case of strain CVM35942. However, strain CVM29188 did not exhibit the same effects compared to its plasmid-cured derivative, both in terms of virulence in this model and colonization in the competition model. Further analysis of CVM29188 via PFGE revealed that its restriction profile was much different than that of the prominent avian *S*. Kentucky PFGE profile (and that of strain CVM35942). Also, strain CVM29188 grew poorly in LB broth compared to CVM35942 and other wild type *S*. Kentucky although this strain performed well in 100% chicken serum. Also, we were unable to find this PFGE profile among the 293 *S*. Kentucky isolates examined. Strain CVM29188 was originally used in this study because it was the sequenced *S*. Kentucky type strain isolated from retail poultry meat identified as harboring a ColV plasmid [12]. However, during the course of this study it became apparent that this isolate was not representative of other typical chicken-associated *S.* Kentucky isolates. The disparate results observed for strain CVM29188, compared to the typical chicken-associated *S*. Kentucky clone, could be attributed to an increased fitness cost for this particular isolate harboring pCVM29188_146, or to changes that might have occurred to its genome during isolation or passage. Nevertheless, it is likely that such changes in strain CVM29188′s genome, compared to CVM35942, have reduced its suitability for persistence in poultry and do not represent what has been identified in this study as a typical poultry-adapted *S*. Kentucky strain.

Despite the apparent emergence of *S*. Kentucky in poultry and its recent increased association with human disease, little is known about the biology of this *Salmonella* serovar. Identification of the ColV plasmid within its genome provides a possible explanation for the clonal expansion and persistence of this *S*. Kentucky clonal type amongst poultry and poultry meat products. While this study provides evidence for some traits that can be attributed to the ColV plasmid in *S*. Kentucky strains, certainly other properties of *S*. Kentucky contribute to its success in poultry, and some of these properties might also be attributed to ColV plasmid carriage. For example, Joerger et al. [37] compared *Salmonella* isolates belonging to different serovars for their virulence gene content, invasiveness towards chicken embryo hepatocytes and human HCT-8 cells, biofilm formation, stress survival, and acid susceptibility. They found that isolates from the *S*. Kentucky serovar differed from other serovars only in their response to acidic growth conditions. Initially *S*. Kentucky responded worse to acidic conditions than other serovars; over the course of 24 hrs *S.* Kentucky grew better than other serovars. The authors speculated that the improved relative growth of *S*. Kentucky in acidic conditions was correlated with it *not* mounting an initially strong and energy-consuming adaptive acid response, thus conserving energy and ultimately growing better than strains that mounted a strong initial response [37]. However, it has also previously been shown that the presence of ColV plasmids in *E. coli* make them more sensitive to killing in acidic conditions [38], thus the observations by Joerger et al. could also be attributed to possession of the ColV plasmid.

Overall, this work suggests that ColV plasmids have been acquired by multiple avian-source *Salmonella* serovars and are particularly prominent among a single clonal type of *S*. Kentucky. Specifically, the acquisition of the ColV plasmid by this clonal lineage of *S*. Kentucky has provided it with an additional repertoire of traits enhancing its ability to persist in poultry. Documented examples of the capture of such “moments in time” in nature involving horizontal gene transfer, resulting in significant shifts in microbial populations, are rare. The impact of this particular transfer event are not yet fully realized, nor is the potential for *S*. Kentucky to acquire additional plasmids and mobile genetic elements. Furthermore, the exact mechanisms of the persistence of *S*. Kentucky in poultry, and the identification of ways to reduce or eliminate this serovar, remain to be determined.

## Methods

### Bacterial strains

ColV plasmid-containing and plasmid-lacking strains were used for fitness, colonization, and virulence assays (Table 3). Some of these strains have been previously described [12]. Additionally, 902 poultry-source *Salmonella* isolates representing 59 serovars, serotyped at the USDA National Veterinary Services Laboratories, were screened for the presence of ColV plasmid-associated genes, as described below. These isolates came from a number of chicken and turkey farms or processing facilities within the United States. Isolates included those from feed, water, darkling beetles (*Alphitobius diaperinus*), transport crates, carcass rinses, litter samples, drag swabs, cloacal swabs, boot socks and diagnostic sources. *Salmonella* isolation was performed as previously described [39].

### ColV plasmid prevalence among avian-source *Salmonella* isolates

Multiplex PCR was used to determine the prevalence of ColV plasmid-associated genes among *Salmonella* isolates using ColV plasmid-associated primer sets and internal control primers for *Salmonella* spp. (Table 1) [40]. An isolate was considered to possess the ColV plasmid if it contained *cvaC* plus additional plasmid-associated genes.

### Bacterial growth curves

Spontaneous mutants with nalidixic acid or rifampicin resistance were created by growing wild type strains overnight in 5 mL Luria-Bertani (LB) broth (BD Biosciences, San Jose, CA), pelleting cells, resuspending in 200 µL of fresh LB broth and plating on MacConkey agar containing 30 µg/mL nalidixic acid or 100 µg/mL rifampicin, respectively. To verify that mutations conferring nalidixic acid or rifampicin resistance did not affect the growth rates of the *S*. Kentucky isolates, and to compare plasmid-containing and plasmid-lacking *S*. Kentucky isolates for their relative *in vitro* growth capabilities, the growth rates of selected *Salmonella* isolates were compared in LB broth. Strains were grown overnight in a 2 ml culture of LB broth. The next day, overnight cultures were pelleted, washed in phosphate-buffered saline (PBS), and diluted in fresh LB broth to a starting OD_600_ of 0.05. Cultures were incubated in 96-well plates at 37°C with shaking in a SpectraMax Plus^384^ spectrophotometer. Samples were taken every 15 minutes for 18 hours. Two independent trials were performed with four replicates per trial. In all of the described growth assays, the inoculum was limited to less than 5% of the total volume to limit dilution of the growth media used. Growth media tested included LB broth, LB broth plus 2,2′-dipyridyl, 50% filtered human serum in LB broth (Life Technologies, Carlsbad, CA), and 100% filtered chicken serum (Sigma-Aldrich Corporation, St. Louis, MO).

### Pulsed-field gel electrophoresis

Spontaneous mutants, plasmid-cured derivatives, and transconjugants were also compared to their wild type counterparts by PFGE using the PulseNet one-day (24–28 h) standardized laboratory protocol [12]. Additionally, a subset of 71 *S*. Kentucky isolates from chickens were analyzed via this procedure. *S.* Branderup H9812 (ATCC#: BAA-664) was used as the size standard. Restriction was carried out using *Xba*I (Roche Applied Science, Indianapolis, IN). DNA macrorestriction fragments were resolved on 1% SeaKem Gold Agarose (Cambrex Bio Science Rockland, Inc., ME, USA) in 0.5× Tris-Borate EDTA. Images were captured using a UV imager (Cell Biosciences, Santa Clara, CA) and stored as tif files. Macrorestriction patterns were compared using the BioNumerics software (Version 5.1; Applied Maths, Inc., Austin, TX). The similarity index of the isolates was calculated using the Dice correlation coefficient option of the software with a position tolerance of 1% and an optimization of 5%. The unweighted-pair group method using average linkages (UPGMA) was used to construct a dendrogram.

### Comparative plasmid sequencing

Total plasmid DNA for visualization and pyrosequencing was isolated from *S*. Kentucky strains CS0010-A and SSAP03002A with the Qiagen Midi Plasmid Kit, using a modified procedure optimized for BAC isolation (http://www.qiagen.com). A single colony was inoculated into 100 ml of LB broth and grown overnight at 37°C with shaking. After purification, plasmid DNA was precipitated twice with three volumes of 100% ethanol, washed twice with 70% ethanol, and dissolved in sterile water. Pyrosequencing of each plasmid was performed using the Roche 454 GS-FLX sequencer with Titanium chemistry at the University of Minnesota's Biomedical Genomics Center, as previously described [41]. Plasmid sequences are deposited in Genbank under the accession numbers CP002089 and CP002090.

### Chicken model of relative colonization and invasion

All animal experiments were performed in accordance with the Institutional Animal Care and Use Committee at the University of Minnesota, approved by the review committee board under protocol number 0708A15286. A chicken model of relative colonization and invasion was used to assess the effects of ColV plasmid acquisition, or loss, on the ability of *Salmonella* strains to colonize the chicken cecum and invade the spleen [42], [43]. Specific-pathogen-free white leghorn eggs were purchased from HyVac (Adel, IA) and hatched at the University of Minnesota's Poultry Isolation Facility, and placed in isolation chambers. Bacterial strains were grown to mid-log phase in LB broth and resuspended in sterile PBS. Bacterial suspensions were administered to 1-week-old chickens at a volume of 0.5 ml and quantity of 1×10^8^ CFU. Each experimental group contained 12 birds. Food and water were fed to the birds *ad libitum*. At 3, 7, and 14 days post-inoculation, four birds were removed from each experimental group and humanely euthanized. The ceca and spleen were aseptically removed from each bird and homogenized in PBS. Ten-fold serial dilutions were performed and plated onto MacConkey agar containing selective antibiotics. Colonies were counted from each plate to determine CFU/g of tissue. Additionally, PCR was performed on recovered colonies to ensure that the original test strain was recovered. Data was statistically analyzed using a one-way analysis of variance (ANOVA) and Tukey's range test.

### Chicken competition model of colonization

A chicken model of colonization was also used to compete ColV plasmid-containing and plasmid-lacking *Salmonella* strains against one another [42], [43]. Three competitions were performed: 1) *S*. Kentucky CVM35942 vs. *S*. Kentucky CVM35942p; 2) *S*. Kentucky CVM29188 vs. *S*. Kentucky CVM29188c; and 3) *S*. Kentucky CVM35942p vs. *S*. Newport SL317. Experiments were performed similar to the colonization model described above, except that equal concentrations of competing strains with different selectable markers were combined to a final concentration of 1×10^8^ CFU. Each experimental group contained 12 birds. At 3, 7, and 14 days post-inoculation, 4 birds were removed from each experimental group, euthanized, their ceca collected and homogenized in PBS, and dilutions plated on appropriate selective media as described above. Competing strains were oppositely labeled with nalidixic acid or rifampicin and plated with and without tetracycline in both cases to ensure that conjugation of the plasmid had not occurred *in vivo*. The ratio of the CFU/g tissue of the two strains recovered from cecal pouches was determined and compared to the input ratios to determine the competitive indices (CI) for each competition. The geometric means of the CIs were determined. Log-transformed ratios were compared using a Student's *t-*test to determine if the values differed significantly (P<0.05) from a value of 1 [42].

### Chicken model of extraintestinal virulence

A generalized subcutaneous model of infection was used in broiler chicks to study the ColV plasmid's contributions to extraintestinal fitness and virulence [44], [45]. Day-old broiler chicks, vaccinated against Marek's disease, were obtained from a commercial source and were divided into groups of 10 birds each in poultry isolation chambers. Chicks were provided food and water *ad libitum*. Birds were injected subcutaneously with 0.1 mL of a bacterial suspension containing 1×10^7^ CFU/ml of bacteria or with 0.1 ml of sterile PBS in the back of the neck. Chicks were challenged on the day after they were received and were subsequently monitored for the first 6 hours post-challenge on day 1, then every 12 hours for 7 days. Deaths were recorded, and the survivors were euthanatized and examined for macroscopic lesions. Organisms were compared to wild type positive control strains APEC O1 (20) and APEC O2 [17] and negative bacterial control strain DH10B. A scoring system (0–12) was used as previously described [46] and was based upon lesions in the air sacs, liver, and pericardium, and on bacterial recovery from these organs. This experiment was repeated and the data were combined and analyzed using the Wilcoxon signed-rank test [47].

## Supporting Information

Table S1 **Prevalence of ColV plasmid-associated genes among** ***Salmonella*** **isolates.**
(DOC)

Table S2 **Prevalence of ColV plasmid-associated genes among** ***S*** **. Kentucky isolates.**
(DOC)
