# Supplementary material for: Horizontal Gene Transfer of a ColV Plasmid Has Resulted in a Dominant Avian Clonal Type of Salmonella enterica Serovar Kentucky
Source: PLoS One. 2010 Dec 22;5(12):e15524. doi: 10.1371/journal.pone.0015524 (PMC3008734; doi:10.1371/journal.pone.0015524)
Supplement: Table S1 — Prevalence of ColV plasmid-associated genes among Salmonella isolates. (DOC) [file pone.0015524.s001.doc]

Table S1. Prevalence of ColV plasmid-associated genes among *Salmonella* isolates.

| Serovar | N | Source(s) | Percent positive for ColV plasmid |
| --- | --- | --- | --- |
| 4,[5],12:i:- | 2 | Chicken | 0.0 |
| Abortusequi | 1 | Turkey | 0.0 |
| Albany | 4 | Turkey | 0.0 |
| Anatum | 1 | Turkey | 0.0 |
| Arizona | 1 | Turkey | 0.0 |
| Bareilly | 2 | Turkey | 0.0 |
| Berlin | 1 | Turkey | 0.0 |
| Berta | 9 | Turkey | 0.0 |
| Binza | 1 | Turkey | 0.0 |
| Blockley | 1 | Turkey | 0.0 |
| Brandenburg | 1 | Turkey | 0.0 |
| California | 1 | Turkey | 0.0 |
| Cambridge | 3 | Turkey | 0.0 |
| Chameleon | 5 | Turkey | 0.0 |
| Chester | 1 | Turkey | 0.0 |
| Chittagong | 3 | Turkey | 0.0 |
| Cubana | 1 | Chicken | 0.0 |
| Daressalaam | 2 | Turkey | 0.0 |
| Drypool | 2 | Turkey | 0.0 |
| Dublin | 10 | Turkey | 0.0 |
| Durban | 2 | Turkey | 0.0 |
| Eastbourne | 3 | Turkey | 0.0 |
| Eimsbuettel | 1 | Turkey | 0.0 |
| Enteriditis | 29 | Chicken and turkey | 0.0 |
| Fresno | 6 | Turkey | 0.0 |
| Gallinarum | 2 | Turkey | 0.0 |
| Gaminara | 2 | Turkey | 0.0 |
| Glostrup | 2 | Turkey | 0.0 |
| Hadar | 5 | Turkey | 0.0 |
| Havana | 2 | Turkey | 0.0 |
| Heidelberg | 119 | Chicken, turkey, and retail meats | 1.7 |
| Infantis | 1 | Chicken | 0.0 |
| Javiana | 8 | Turkey | 0.0 |
| Jordana | 1 | Turkey | 0.0 |
| Kentucky | 293 | Chicken and turkey | 72.7 |
| Kiambu | 3 | Chicken | 0.0 |
| Lille | 3 | Turkey | 0.0 |
| Livingstone | 1 | Turkey | 0.0 |
| Miami | 12 | Turkey | 0.0 |
| Moscow | 3 | Turkey | 0.0 |
| Newport | 144 | Chicken and Turkey | 0.0 |
| Ohio | 2 | Chicken | 0.0 |
| Oranienburg | 1 | Turkey | 0.0 |
| Panama | 10 | Turkey | 0.0 |
| Pomona | 2 | Turkey | 0.0 |
| Poona | 3 | Turkey | 0.0 |
| Portsmouth | 3 | Turkey | 0.0 |
| Pullorum | 17 | Chicken and Turkey | 0.0 |
| Quakam | 4 | Chicken | 0.0 |
| Schwarzengrund | 28 | Chicken and turkey | 0.0 |
| Senftenberg | 1 | Turkey | 0.0 |
| Taksony | 2 | Turkey | 0.0 |
| Tennessee | 2 | Chicken and turkey | 0.0 |
| Thomasville | 1 | Turkey | 0.0 |
| Thompson | 2 | Chicken | 0.0 |
| Typhimurium | 100 | Chicken, turkey, and retail meats | 15.0 |
| Urbana | 3 | Turkey | 0.0 |
| Westhampton | 2 | Turkey | 0.0 |
| Worthington | 25 | Chicken and turkey | 0.0 |
|  | 902 |  | 29.9 |
