# Supplementary material for: Horizontal Gene Transfer of a ColV Plasmid Has Resulted in a Dominant Avian Clonal Type of Salmonella enterica Serovar Kentucky
Source: PLoS One. 2010 Dec 22;5(12):e15524. doi: 10.1371/journal.pone.0015524 (PMC3008734; doi:10.1371/journal.pone.0015524)
Supplement: Table S2 — Prevalence of ColV plasmid-associated genes among S . Kentucky isolates. (DOC) [file pone.0015524.s002.doc]

Table S2. Prevalence of ColV plasmid-associated genes among *S*. Kentucky isolates.

| **Sample type** | **N** | **Percent positive for ColV plasmid** |
| --- | --- | --- |
| Litter sample | 24 | 83.3 |
| Drag swab | 37 | 100.0 |
| Cloacal swab | 24 | 95.8 |
| Boot sock sample | 78 | 75.6 |
| Processing plant sample | 50 | 38.0 |
| Other | 84 | 70.2 |
| Overall | 297 | 72.9 |
